# YBX2 and cancer testis antigen 45 contribute to stemness, chemoresistance and a high degree of malignancy in human endometrial cancer

**DOI:** 10.1038/s41598-021-83200-5

**Published:** 2021-02-18

**Authors:** Izumi Suzuki, Sachiko Yoshida, Kouichi Tabu, Soshi Kusunoki, Yumiko Matsumura, Hiroto Izumi, Kazuo Asanoma, Hiroshi Yagi, Ichiro Onoyama, Kenzo Sonoda, Kimitoshi Kohno, Tetsuya Taga, Atsuo Itakura, Satoru Takeda, Kiyoko Kato

**Affiliations:** 1 Department of Obstetrics and Gynecology, Faculty of Medicine, Juntendo University, 2-1-1 Hongo, Bunkyo-ku, Tokyo, 113-8431 Japan; 2 Department of Obstetrics and Gynecology, Graduate School of Medical Sciences, Kyushu University, 3-1-1 Maidashi, Higashi-ku, Fukuoka, 812-8582 Japan; 3 Department of Stem Cell Regulation, Medical Research Institute, Tokyo Medical and Dental University (TMDU), 1-5-45 Yushima, Bunkyo-ku, Tokyo, 113-8510 Japan; 4 Department of Occupational Pneumology, Institute of Industrial Ecological Science, University of Occupational and Environmental Health School of Medicine, 1-1 Iseigaoka, Yahatanishi-ku, Kitakyushu, Fukuoka 807-8555 Japan; 5 Gynecology Service, National Kyushu Cancer Center, Fukuoka, 3-1-1 Notame, Minami-ku, Fukuoka, 811-1395 Japan; 6 Kurate Hospital, 2425-9 Ooaza Nakayama, Kurate-chou, Kurate, Fukuoka 807-1312 Japan

**Keywords:** Endometrial cancer, Cancer stem cells

## Abstract

Y-box binding protein 2 (YBX2) has been associated with the properties of both germ cells and cancer cells. We hypothesized that YBX2 might contribute to the characteristics of cancer stem cells (CSCs). In this study, we clarified the function of YBX2 in endometrial cancer stem cells. We established a human YBX2-expressing Ishikawa (IK) cell line (IK-YBX2 cells). We analyzed gene expression associated with stemness and isolated SP cells from IK-YBX2 cells. The SP population of IK-YBX2 cells, the expression of *ALDH1* and serial sphere-forming capacity were associated with levels of YBX2 expression. IK-YBX2 cells were resistant to anti-cancer drugs. In gene expression analysis, a gene for cancer testis antigen, *CT45*, was generally overexpressed in IK-YBX2 cells. YBX2-mediated CT45 expression was associated with increased levels of self-renewal capacity and paclitaxel resistance. The level of CT45 expression was enhanced in high-grade and/or advanced stages of human endometrial cancer tissues. We conclude that expression of YBX2 is essential for the stem cell-like phenotype. CT45 contributes to stemness associated with YBX2 and might be related to the progression of endometrial cancer.

## Introduction

Endometrial cancer is the most common gynecological cancer for women in high-income countries and incidences are increasing^1,2^. Whereas prognoses of patients with low-grade and early-stage endometrial cancer are good, the outcomes of those with high-grade and metastatic or recurrent cancer remain poor with current diagnostic methods and treatments, like poor stratification into treatment regimens and lack of targeted therapy approach^3^. To eliminate cancer in patients with poor prognoses, we need to achieve more knowledge on resistance mechanisms and develop better markers and treatment strategies to combat this.

CSCs constitute a small subpopulation of cells within tumors with the capacity for self-renewal and multi-potential differentiation^4,5^. Recent studies have suggested that CSCs are present in several types of malignant tumors, such as leukemia^6,7^, breast cancer^8^ and brain tumors^9^. CSCs have properties such as quiescence, high activity of several ATP-binding cassette transporters (ABC transporters), an active DNA-repair capacity and anti-apoptosis activity. Therefore, they are often resistant to chemotherapy and are able to facilitate the regrowth of tumors^10^.

There are several methods that can be used to identify CSCs. CD133, CD44 and aldehyde dehydrogenase 1 (ALDH1) are enriched in CSCs and are used for their identification^11^. Side-population (SP) cells, a stem cell-enriched subpopulation, are identified by flow cytometry based upon their ability to pump out intracellular Hoechst 33342^11,12^. Elevated expression of ABCG2/BCRP1, the ABC transporter protein, is associated with the SP phenotype^13^. Established malignant cell lines retain SP cells as a minor subpopulation even after long-term culture^14^. We have isolated SP cells from a human endometrial cancer cell line (Hec-1 cells) and rat endometrial cells expressing oncogenic human K-Ras protein (RK12V cells). We have previously showed that SP cells of endometrial cancer show CSC characteristics^15,16^.

Y-box-binding proteins belong to the human cold-shock domain protein superfamily. They include dbpC/Y-box-binding protein 2 (YBX2), Y-box binding protein 3 (YBX3), and dbpB/YB-1. Y-box-binding proteins play a role in a variety of environmental stress reactions such as responses to low temperatures. Cold shock proteins (CSP), which are induced by low temperatures, control transcriptional and translational processes. These proteins act as a basic defense system^17^. YBX2 is a germ cell-specific Y-box-binding protein. It is expressed in the germ cells of adult testes and in developing fetal testes and ovaries, but not in any other normal tissues^18,19^. The function of YBX2 includes the storage and translation of mRNAs in germ cells. The *YBX2* gene is expressed in early embryogenesis. However, little is known about its function. Kohno et al. reported that YBX2 is expressed in the placenta, in germ cell tumors and various human carcinomas^17^. Its restricted pattern of expression suggests that it might be associated with cancer/testis antigens (CTAs).

CTAs are human tumor antigens. CTAs are expressed in various types of human cancer, whereas they are restricted to normal germ cells^20^. Therefore, CTAs are markers for diagnoses and targets for therapies in cancer^21^. Moreover, several CTAs are expressed at high levels in CSC-like populations isolated from various tumor types^22,23^. Because the restricted expression pattern of YBX2 is similar to CTAs associated with the CSC phenotype, we hypothesized that YBX2 might contribute to the characteristics of CSCs. Here, we have analyzed YBX2 function and YBX2-induced genes.

## Results

### YBX2 was expressed focally in human endometrial cancer tissues: expression levels were elevated in SP cells

The characteristics of patients in the present study are shown in Supplementary Table S1 online. Endometrioid carcinomas were defined as follows: well-differentiated, grade 1; moderately differentiated, grade 2; poorly differentiated grade 3. First, we confirmed YBX2 protein expression in human endometrial cancer tissues. The entire slide was evaluated with the Allred scoring system in 2 categories (stain intensity and stain pattern) (Supplementary Table S2 online). YBX2 was expressed focally (Supplementary Fig. S1a online) and the number of positive cases with the Allred scoring system was higher in grade 3 than grade 1 (Supplementary Table S3 online). These results demonstrated that YBX2 was expressed in a higher proportion of cancer tissues in high grade cases than low grade cases.

Next, to confirm the contribution of *YBX2* gene expression in SP cells, we investigated *YBX2* expression levels in Hec1 SP cells and non-SP (NSP) cells by real-time PCR. The level of *YBX2* gene expression was enhanced in Hec1 SP cells compared with that in Hec1 NSP cells (Supplementary Fig. S1b online).

### Forced expression of YBX2 contributed to increases in the stem-like cell population

**Figure 1:**
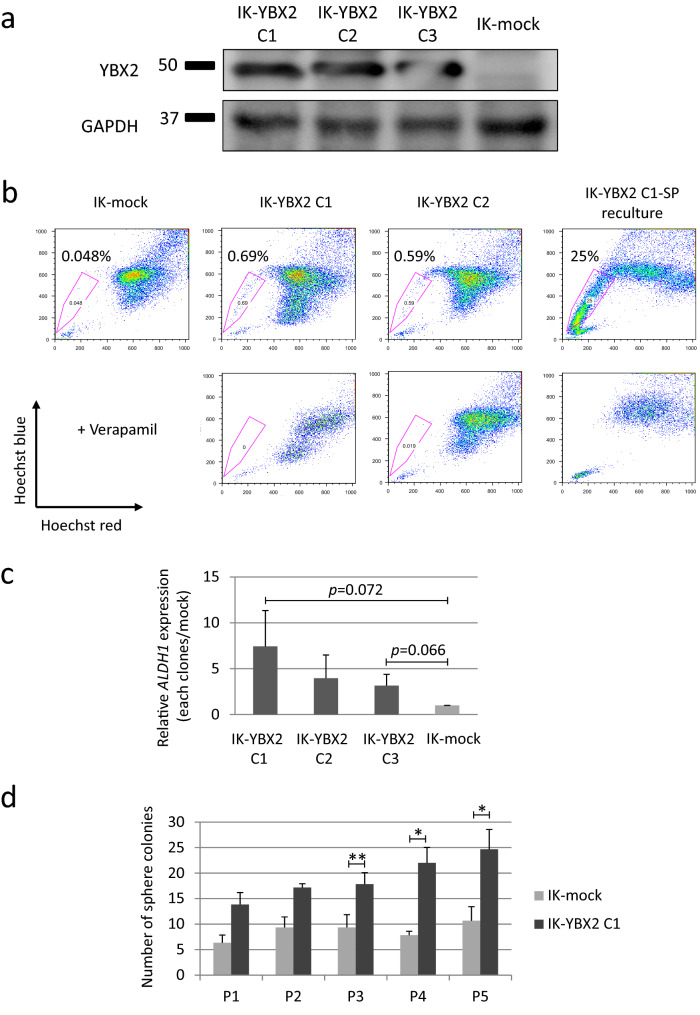
The expression of YBX2 contributed to the characteristics of cancer-stem cells. (**a**) YBX2-overexpressing Ishikawa (IK) cell lines were established by transfection with the pcDNA3 vector containing *YBX2* cDNA. YBX2 protein expression in three established IK-YBX2 cells was confirmed by Western blotting. Full length blots are shown in Supplementary Fig. S5a online. (**b**) SP cells were isolated from two IK-YBX cell lines (C1 and C2) and mock cells. SP cells from IK-YBX2 C1 cells were cultured for two weeks and re-analyzed by flow cytometry. Similar results were obtained from independent experiments on another clones. (**c**) The expression of *ALDH1* in all three IK-YBX2 cells (each clone: C1, C2, C3) and mock cells was investigated by real-time PCR. *GAPDH* level was used as internal standards. Relative ratios are represented as the means ± SD from three independent experiments (C1 *p* = 0.072, C2 *p* = 0.119, C3 *p* = 0.066). (**d**) The serial spheres formation assays were performed using IK-YBX2 C1 cells and IK-mock cells. Sphere formation was counted at passage (P) 1 to 5. The serial sphere forming capacity, was enhanced in IK-YBX2 C1 cells compared with in IK-mock cells. (P3: **p < 0.01, P4, P5: *p < 0.05).

IK cell populations include few SP cells. To confirm the effect of *YBX2* expression on the frequency of SP cells, we established IK cells that overexpressed YBX2 (IK-YBX2 cells) as described in “Methods”. Three different clones were established (Fig. 1a). We analyzed the proportion of SP cells in two IK-YBX2 cell lines (C1 and C2) and in mock cells. The populations of SP cells were larger in both clones of IK-YBX2 cells compared with mock cells (C1, 0.69%, C2, 0.59% and mock 0.048%) (Fig. 1b). Then, SP cells and NSP cells from IK-YBX2 C1 cells were cultured. SP cells from the IK- YBX2 cells (IK-YBX2 SP cells) grew for at least 8 weeks. In contrast, NSP cells from the cultures stopped growing within 2 weeks (data not shown). These results were consistent with our previous data with Hec1-SP cells and -NSP cells^15^. Next, SP cells were isolated from IK-YBX2 cells that had been cultured for two weeks and then re-analyzed again by flow cytometry. Intriguingly, the proportion of SP cells was remarkably enhanced (25%) (Fig. 1b).

The level of *ALDH1*, which is a marker for both normal stem cells and cancer stem cells^24^, was investigated by real-time PCR. The levels of *ALDH1* expression were increased in all 3 IK-YBX2 clones compared with that in mock cells, however the differences were not significant (C1 *p* = 0.072, C2 *p* = 0.119, C3 *p* = 0.066) (Fig. 1c). Next, we performed serial sphere formation assays, which shows self-renewal potential^9^. We used IK-YBX2 C1 cells and IK-mock cells. Sphere formation was counted at passages (P) 1 to 5. The serial sphere forming capacity was enhanced in IK-YBX2 cells compared with IK-mock cells (Fig. 1d).

### The knockdown of YBX2 reduced SP cells and ALDH1

**Figure 2:**
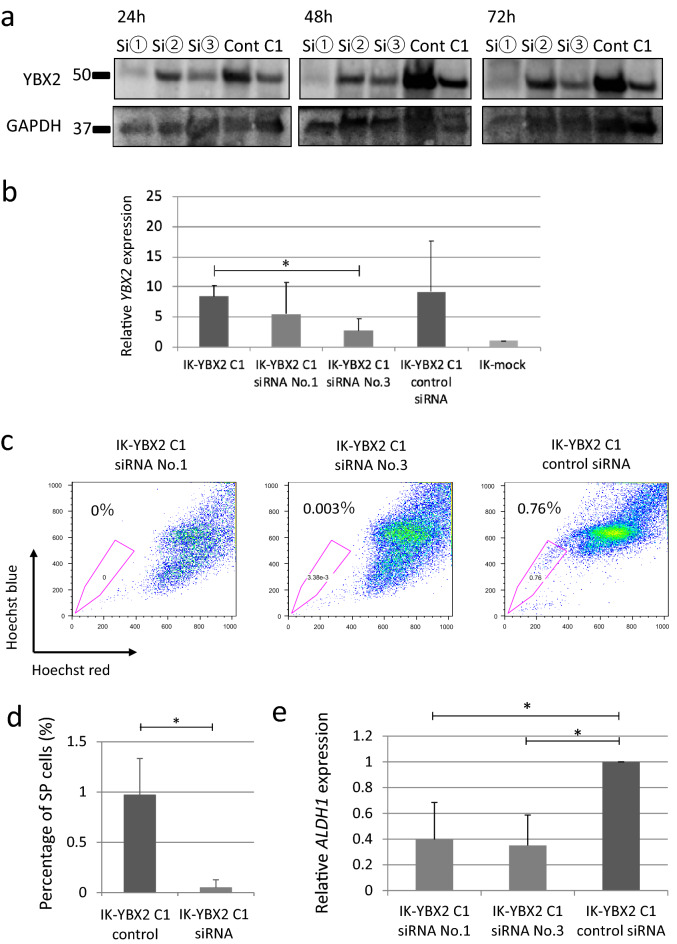
The effect of the knockdown of YBX2. (**a**) The level of YBX2 protein expression was investigated by Western blotting. Full length blots are shown in Supplementary Fig. S5b online. (**b**) The level of *YBX2* gene expression using two siRNAs (No.1 or No.3) was investigated by real-time PCR. *GAPDH* level was used as internal standards. Relative ratios are represented as the means ± SD from three independent experiments (*p < 0.05). (**c**) Downregulation of YBX2 protein using two siRNAs led to significant decreases in the population of SP cells compared with that using control siRNA. (**d**) The percentage of SP cells was decreased in IK-YBX2 C1 siRNAs cells compared with IK YBX2 C1 control siRNA cell from three independent experiments (*p < 0.05). (**e**) The level of *ALDH1* expression was decreased in IK-YBX2 C1 siRNAs No.1 and No.3 compared with IK-YBX2 C1 control siRNA cell. *HPRT* level was used as internal standards (*p < 0.05).

Next, we knocked down *YBX2* gene expression in IK-YBX2 C1 cells using 3 different types of *YBX2*-siRNAs described in “Methods”. The levels of YBX2 protein and gene expression were investigated by Western blotting and real-time PCR. Two types of siRNA (No. 1 and No. 3) reduced the expression of YBX2 protein (Fig. 2a). The level of *YBX2* expression was significantly decreased in IK-YBX2 C1 siRNA No. 3 cells (*p* < 0.05) (Fig. 2b). Downregulation of YBX2 using both siRNAs (No. 1 and No. 3) decreased the population of SP cells compared with that using control siRNA (siRNA No. 1, 0%, siRNA No. 3; 0.003%, control siRNA 0.76%) (representative data Fig. 2c). The percentage of SP cells in IK-YBX2 C1 cells was lower than that in YBX2 in 3 independent experiments (*p* < 0.05) (Fig. 2d). Moreover, the levels of *ALDH1* expression were significantly decreased in cells treated with IK-YBX2 C1 siRNA No. 1 and No. 3 compared with IK-YBX2 C1 control siRNA cells (*p* < 0.05) (Fig. 2e).

### YBX2 suppressed cell growth in vivo, and kept the cell cycle at the G0/G1 phase transition

Stem cells show a slow growing phenotype^25^. We subcutaneously injected IK-YBX2 C1 and C3 cells or mock cells into the dorsal hypodermis of nude mice. We found that the tumor sizes from IK-YBX2 was smaller than those generated by mock cells (Supplementary Fig. S2a online). To clarify the mechanism of lower growth rate, we performed cell cycle analysis. Flow cytometric analyses revealed that the ratio of the IK-YBX2 C1 cell population in the S phase and G2/M phase did not change until 12 h of culture. On the other hand, the ratio of the IK mock cell population in S phase was significantly increased after 6 h of culture. Furthermore, the fraction of the IK mock cell population in the G2/M phase was also significantly increased at 6 and 12 h (*p* < 0.05) (Supplementary Fig. S2b online). These results demonstrated that YBX2 expression decreased the G0/G1 phase transition of cell cycle, resulting in slow- cycling, which is one of the characteristics of stem cells.

### YBX2 induced CT45A5 expression in endometrial cancer cells

Next, to identify the genes that contributed to increased stemness by *YBX2* expression, we analyzed enhanced gene expression of IK-YBX2 cells and mock cells using microarray analysis. We identified 751 genes that were upregulated more than twofold in IK-YBX2 C1 cells compared with those in IK-mock cells. *CT45A5* was the most upregulated gene in IK-YBX2 C1 cells, compared with mock cells (Supplementary Table S4 online), and the same result was obtained in IK-YBX2 C3 cells and IK-YBX2 C1 SP cells, compared with mock cells.

CT45, a cancer testis antigen, was initially identified using massively parallel signature sequencing (MPSS) in 2005^26^. The CT45 family is comprised of six very similar genes (CT45A1 to CT45A6) that are clustered in tandem on Xq26.3.

**Figure 3:**
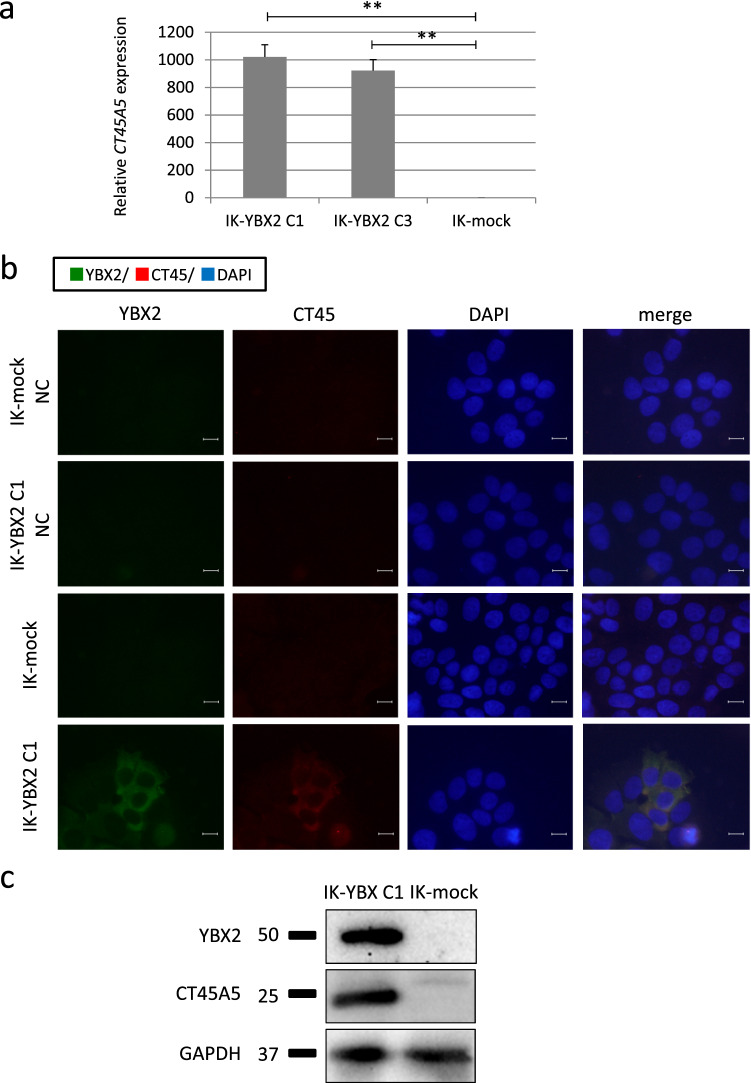
The levels of *CT45A5* gene and protein expression were significantly increased in IK-YBX2 cells compared with those in mock cells. (**a**) The level of *CT45A5* expressed in IK-YBX2 cells (C1 and C3) was increased more than 900-fold compared with that in mock cells. *GAPDH* was used as internal control. Data represent the means ± SD from three independent experiments. (**p < 0.01). (**b**) YBX2 and CT45 expression levels in IK-YBX2 C1 cells were detected by fluorescence microscopy. Co-expression of YBX2 and CT45 in the cytoplasm of IK-YBX2 C1 cells was observed. Scale bars: 10 µm. (**c**) The levels of both proteins of YBX2 and CT45 were markedly increased in IK-YBX2 C1 cells and compared with that in mock cells. Full length blots are shown in Supplementary Fig. S5c online.

By using real-time PCR, we assessed the level of *CT45A5* mRNA. *CT45A5* expression in IK-YBX2 cells (C1 and C3) was increased more than 900-fold compared with that in mock cells (C1, C3: *p* < 0.01) (Fig. 3a). Next, we investigated YBX2 protein expression. We monitored co-expression of YBX2 and CT45 in IK-YBX2 cells by immunofluorescence staining using an antibody detecting CT45. We observed co-expression of YBX2 and CT45 in the cytoplasm of IK-YBX2 C1 cells (Fig. 3b). Finally, we stained for an antibody detecting CT45A5. Western blotting analysis showed that the expression of CT45A5 was markedly increased in IK-YBX2 C1 cells compared with that in mock cells (Fig. 3c).

### CT45A5 expression was critical for an increase of self-renewal capacity in IK-YBX2 cells

**Figure 4:**
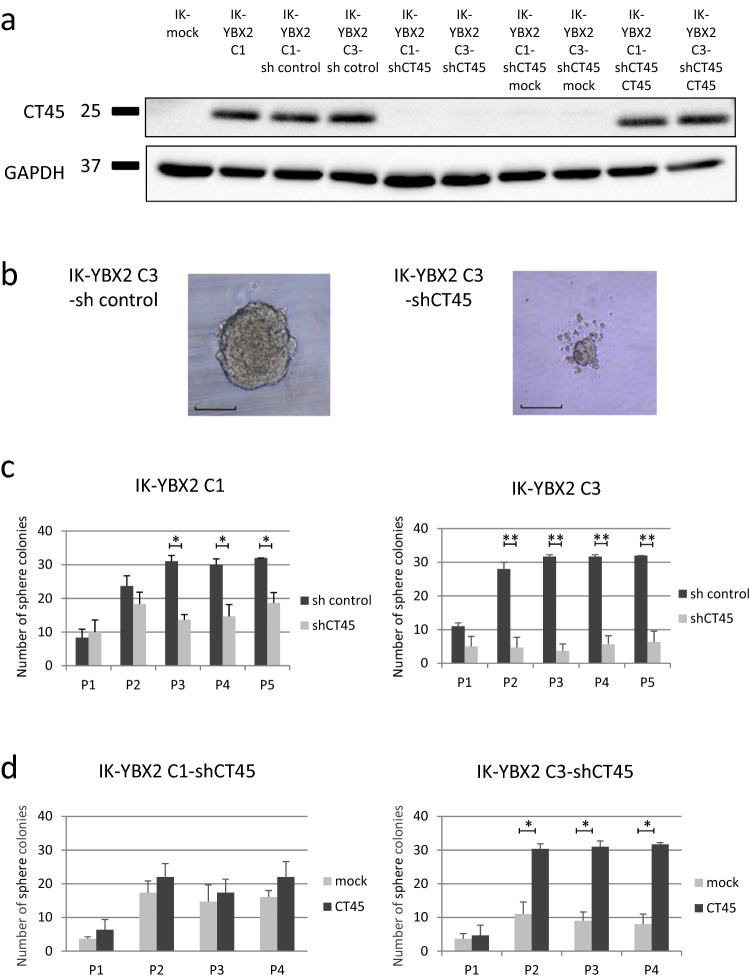
Effects of CT45 expression on self-renewal in endometrial cancer cell lines. To investigate of CT45 expression, stable knockdown of CT45 of IK-YBX2 cells and *CT45* gene-rescued cells of them were established. (**a**) The level of CT45 expression of established cells was investigated by Western blotting. shRNAs significantly reduced the expression of CT45 (lane 3, 4 vs lane 5, 6), and the proteins were rescued using relevant gene expression (lane 7, 8 vs lane 9, 10). Full length blots are shown in Supplementary Fig. S5d online. (**b**) The serial spheres formation assays were performed using two different clones of IK-YBX2-shCT45 and IK-YBX2-sh control cells respectively. Representative spheres generated from a single cell after 14 days in non-adherent culture were shown (scale bar 150 μm). (**c**) Sphere formation was counted at passage (P) 1 to 5. The serial sphere forming capacity, was reduced in IK-YBX2-shCT45 cells compared with in IK-YBX2-sh control cells (**p < 0.01, *p < 0.05). (**d**) To exclude off target effect of shRNA, we also established cells which were rescued CT45 expression by introduction of *CT45* cDNA into each clone of CT45 knocked down cells. Upon serial passage from P1-P4, IK-YBX2-shCT45-mock cells generated few tumorsphere during passages. Conversely, sphere formation efficiency was increased in IK-YBX2-shCT45-CT45 rescued cells and a significant increase was observed in of IK-YBX2 C3-shCT45-CT45 rescued cells (*p < 0.05). Data represent the means ± SD from three independent experiments.

In order to analyze the function of *CT45A5*, we knocked down *CT45A5*, which was overexpressed in IK-YBX2 cells, using 3 untranslated region (*3′-UTR*) shRNAs as described in Supporting information (Supplementary Table S5 online). To exclude off-target effects of shRNA, we also established cells which were rescued for CT45 by introducing *CT45* cDNA without *3′-UTR* into CT45 knockdown cells (Fig. 4a). Suppression was achieved with a shRNA technique as described in “Methods”. We performed serial sphere forming assays. Representative IK-YBX2-shCT45 and sh control spheres generated from a single cell after 14 days in non-adherent culture are shown (Fig. 4b, scale bar 150 μm). Sh control cells produced typical nonadherent spheres, while shCT45 cells had reduced sphere-forming ability that was less than sh control cells (Fig. 4b). The number of tumorspheres/ 32 wells was counted in 5 consecutive passages. Upon serial passage from P1-P5, sphere formation efficiency gradually increased in sh control cells. Conversely, shCT45 generated few tumorspheres during passage (Fig. 4c). Although similar results were obtained from CT45-rescued C1 and C3, no significant difference was shown in C1. It is not certain whether there are off-target effects in CT45sh (Fig. 4d). These results suggested CT45 contributed to self-renewal and that it was related to YBX2 expression.

### The expression of YBX2 and CT45 increased cells’ resistance to paclitaxel, but not cisplatin or carboplatin

**Figure 5:**
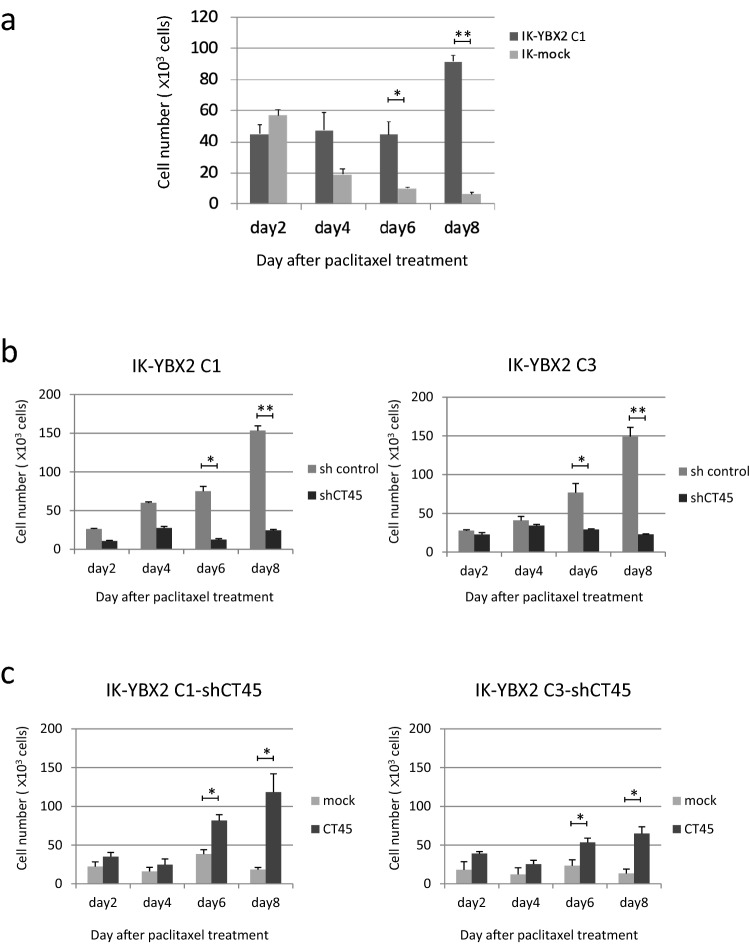
The expression of YBX2 and CT45 caused resistance to paclitaxel. (**a**) Chemosensitivity assay with IK-YBX2 C1 cells and IK mock cells. The number of IK-mock cells decreased over time after treatment with paclitaxel, while that of IK-YBX2 C1 cells was not changed. There were significant differences in the viable cell number at day 6 (*p < 0.05), and day 8 (**p < 0.01). (**b**) Chemosensitivity assay with IK-YBX2 C1 and C3-shCT45 cells and sh control cells was performed. The number of sh control cells increased over time. In contrast, the number of IK-YBX2-shCT45 cells decreased. There were significant differences in the viable cell number at day6 and day8 (*p < 0.05, **p < 0.01). (**c**) Chemosensitivity assay with IK-YBX2-shCT45-CT45 rescued cells and mock cells. Similar to IK-YBX2 cells, CT45-rescued cells displayed the stronger resistance to paclitaxel. There were significant differences in the viable cell number at day6 and day8 (*p < 0.05). Data represent the means ± SD from three independent experiments.

CSCs possess elevated chemo-resistance due to their ability to expel drugs through ABC transporters, the activities of which define the side population phenotype^27^. Furthermore, Rahadiani et al. demonstrated that ALDH1-high expressing cells were more resistant to anti-cancer agents than ALDH1-low expressing cells in endometrial cancers^28^. Therefore, we performed chemosensitivity assays with IK-YBX2 cells and IK mock cells. Doxorubicin plus cisplatin is the standard postoperative adjuvant chemotherapy regimen for endometrial cancer at a high risk of progression. However, in many countries, a combination of paclitaxel and carboplatin therapy (TC therapy) is an alternative postoperative adjuvant regimen for endometrial cancer because of efficacy and tolerability^29^. Therefore, we investigated chemosensitivity of paclitaxel, carboplatin and cisplatin. In the presence of paclitaxel, the number of IK-mock cells decreased over time. In contrast, during treatment with paclitaxel, the number of IK-YBX2 C1 cells was not changed for the first 6 days and actually increased after 8 days. On days 6 and day 8, there was a significant difference in viable cell numbers between the 2 cell groups (day 6: *p* < 0.05; day 8: *p* < 0.01) (Fig. 5a). Thus, the expression of YBX2 elevated the cells’ resistance to paclitaxel.

Next, to investigate the contribution of CT45 to the chemo-resistance of IK-YBX2 cells, we used 2 different clones of IK-YBX2-shCT45 and IK-YBX2-sh control cells in the same assay. Although the number of IK-YBX2-sh control cells increased, the number of IK-YBX2-shCT45 cells did not change over time. There were significant differences in the viable cell number between IK-YBX2-shCT45 and IK-YBX2-sh control cells at day 6 (*p* < 0.05) and day 8 (*p* < 0.01) (Fig. 5b). Furthermore, CT45-rescued IK-YBX2-shCT45 cells displayed greater resistance to paclitaxel. There were significant differences in the viable cell number at days 6 and 8 (*p* < 0.05) (Fig. 5c). We performed the same assay using cisplatin and carboplatin. The results obtained with the clones were different, and the data did not show an association between CT45 levels and platinum-resistance (Supplementary Fig. S3 online). These results suggested that the expression YBX2 and CT45 increased cells’ resistance to paclitaxel, but not cisplatin or carboplatin.

### CT45A5 was overexpressed in high grade endometrial cancer

**Figure 6:**
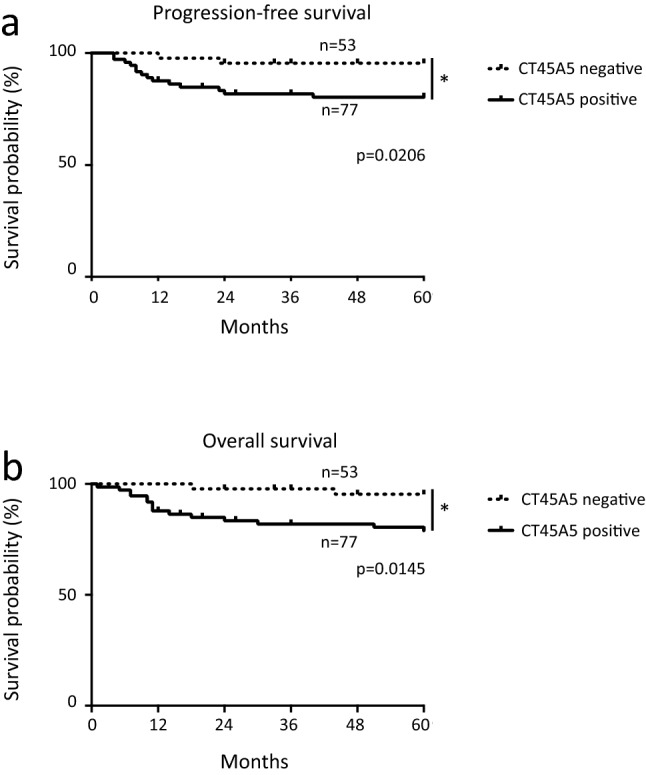
Prognosis analysis of CT45A5 in endometrial cancer tissue was evaluated by the Allred scoring system in which two categories (stain intensity and stain pattern) were evaluated as described in Supplementary Table S2. (**a**) Progression-free survival curves were plotted using the Kaplan–Meier method and compared using the log-rank test. (*p* = 0.0206). (**b**) Overall survival curves were plotted using the Kaplan–Meier method and compared using the long-rank test. (*p* = 0.0145).

Finally, we used immunohistochemistry to investigate the expression of CT45A5 in endometrial cancer tissue. The characteristics of patients used in this study are shown in Supplementary Table S6 online. Samples of endometrial cancer tissue showed several patterns of staining: strong to moderate, partial staining and negative staining (Supplementary Fig. S4 online). The entire slide was evaluated with the Allred scoring system in 2 categories (stain intensity and stain pattern) (Supplementary Table S2 online). Normal endometrium did not express CT45A5. We analyzed the correlation between the expression level of CT45A5 and the histological staging and grade (Table 1). The positive level of CT45A5 in undifferentiated cancer cells in grade 3 (including serous and clear cell histology) was significantly higher than that in the grade 1 group. The level of CT45A5 in advanced cases was significantly higher than that in stage I. Because the number of cases in Stage IV was very low, there was no significant difference between Stage I and Stage IV along with the level of CT45A5. However, 9 of 10 postmortem samples tended to be positive for CT45A5. We conducted a prognosis analysis, and detected significant differences in progression-free survival and overall survival (Fig. 6a, 6b, *p* = 0.0206 and *p* = 0.0145 respectively). These data suggested that the expression of CT45A5 was correlated with high tumor grade and stage.

**Table 1:** Expression pattern of CT45A5 in endometrial cancer tissue.

Grading	Positive	Negative	Positive/total (%)
G1	28	34	45.1
G2	11	11	50.0
G3	17	5	77.2(*)
Clear	10	2	83.3(*)
Serous	11	1	91.6(*)

Staging	Positive	Negative	Positive/total(%)
I	51	46	52.5
II	4	4	50.0
III	21	1	95.4(*)
IV	1	2	33.3
Death cases	8	1	88.8

Expression of CT45A5 in endometrial cancer tissues was investigated by immunohistochemistry. Patient characteristics are shown in Supplementary Table S6 online. The entire slide was evaluated by the Allred scoring system in which two categories (stain intensity and stain pattern) were evaluated as described in Supplementary Table S2. The positive level of CT45A5 in undifferentiated cancer cells involved in G3/serous/clear was significantly higher than that in the G1 group. The level of CT45A5 in advanced cases was significantly higher than that in stage I (*p < 0.05).

## Discussion

In previous reports, the expression of YBX2 has been well described, but little is known about its function. The pattern of YBX2 expression is similar to that of CTAs, and several CTAs are expressed in CSC-like populations^22,23^. Therefore, we hypothesized that YBX2 might contribute to the unique characteristics of stem cells. Because few studies have focused on the relationship between YBX2 and cancer stem cells, we examined how YBX2 contributed to the characteristics of CSCs.

First, we showed that the proportion of SP cells was enhanced in both Hec-1 cells and IK cells that overexpressed YBX2 compared with mock cells. This is the first report demonstrating that the introduction of a single gene increased the proportion of SP cells in an endometrial cancer cell line. Furthermore, the levels of *ALDH1* expression and serial sphere forming activity were enhanced in IK-YBX2 cells compared with IK-mock cells, thereby demonstrating that YBX2 expression is related to stemness.

The proportion of endometrial cancer SP cells was inherently quite small (0.05% in parental IK cells). Thus, it was difficult to identify CSC-specific markers. However, the introduction of YBX2 enhanced the proportion of SP cells, and we were able to perform microarray analysis to identify genes overexpressed in IK-YBX2 cells. We found that the *CT45A5* gene, which is one of the CTAs, was overexpressed in IK-YBX2 cells.　CTAs were initially identified as immunogenic tumor antigens^30^. They are normally expressed in the germ cells of adult testis and developing fetal testis and ovary, but not in any other normal tissue^20,31^. They are also expressed in about 40% of human cancers, such as lung cancer, breast cancer, ovarian cancer, melanoma and urinary bladder carcinoma. Thus far, more than 200 cancer testis antigens have been identified. CT45 is one of the CTAs and 6 types have been identified (CT45A1 to CT45A6)^26^.

In the present study, microarray analysis showed that *CT45A5*, but not other types of *CT45*, was upregulated. CT45 is dynamically expressed during embryonic development and is silenced after birth, but is reactivated in various cancer cell types in a favored tumor microenvironment. CT45 expression has been detected in human cancers, such as lung cancer, ovarian cancer, classical Hodgkin’s lymphoma and diffuse large B-cell lymphoma. In contrast, no evidence of CT45 protein expression has been seen in any non-testicular normal tissue^30^.

In addition, we demonstrated that CT45 was associated with self-renewal ability and anticancer drug-resistance. Shang et al. proposed that overexpression of CT45A1 activated the transcription of multiple oncogenic and metastatic genes, promoted EMT and increased breast cancer cell stemness and invasion^32^. These results suggested that CT45 supports characteristics of CSCs.

CT45 expression is reportedly associated with poor prognosis^31–34^. We demonstrated that CT45 expression was increased in undifferentiated cancer cells and higher clinical stage in endometrial cancer cases. Several CTAs are considered to be targets for cancer immunotherapy because CTAs are expressed in various cancer types whereas their expression is restricted in normal tissues. Cancer vaccine trials based on several CTAs are currently ongoing^35^. CT 45 might be an ideal target in cancer immunotherapy, in particular, for CSCs.

In summary, our study demonstrated that the expression of YBX2 contributed to the stem cell-like phenotype in endometrial cancer stem cells. Moreover, YBX2 induced the expression of cancer testis antigen, CT45, which has the potential for maintaining the stemness of such cells.

## Methods

### Patients and samples

Endometrial cancer patients (130 total: 100 from Juntendo University and 30 from Kyushu University) constituted the participants in the study. All patients underwent surgical resection between 1994 and 2008 and subsequently received radiation therapy and chemotherapy. This study was approved by the bioethics committee of Juntendo University (project number: 21043) and the ethical committee of Kyushu University (project number: 622). This study complied with the guidelines of both universities. All patients gave written informed consent. For death cases Informed consent obtained from a parent and/or legal guardian.

### Immunohistochemistry

Paraffin sections were deparaffinized, and rehydrated. Slides were washed in PBS, and immune-labeled with primary antibodies against CT45 and YBX2 overnight at 4 °C after incubation with Protein Block Serum Free (Dako, Carpentaria, CA) to eliminate nonspecific binding. The slides were then rinsed with PBS and incubated with anti-rabbit secondary antibody (DAKO Envision + Dual Link System HRP; Dako) at room temperature for 1 h. Visualization of the immunoreaction was carried out by incubation with 3,3-diaminobenzidine (DAB). Finally, sections were counterstained with hematoxylin. The antibodies used with dilution and providers are listed in Supplementary Table S7 online. Immunohistochemistry staining was detected under the BZ-X710 microscope (Keyence, Osaka, Japan). The entire slide was evaluated with the Allred scoring system^36^: two categories (stain intensity and stain pattern) were evaluated (Supplementary Table S2 online).

### Plasmids

The pcDNA3vector was purchased from Invitrogen (Carlsbad, CA). The pEGF-*YBX2* expression vector was a gift from Dr. Kimitoshi Kohno. We cut the fragment containing *YBX2* from the pEGF-*YBX2* expression vector with EcoRI and ligated it into the EcoRI site of the pcDNA3 vector.

### Cell lines and cell culture

Two endometrial cancer cell lines, Ishikawa (IK) and Hec-1 were used in this study. IK cells were purchased from Sigma-Aldrich (St. Louis, MO). The Hec-1 cell line was established by Kuramoto et al*.* from a human endometrial adenocarcinoma explant^37^, and were purchased from the JCRB Cell Bank. Cells were grown in DMEM supplemented with 10% fetal bovine serum, penicillin, and streptomycin.

IK cells and Hec-1 cells harboring *YBX2* were established by transfection with the pcDNA3 vector containing *YBX2* cDNA. Transfection was performed with the Amaxa Cell Line Nucleofector Kit L (Lonza, Basel, Switzerland) according to the manufacturer’s protocol as performed before in our study^38^. The cells were transfected by using program T-020 and the sample was transferred to six-well plates. Stably transfected cells were selected and isolated in growth medium containing 400 μg/mL of G418 (Invitrogen).

### Isolation of SP cells

SP cells were isolated as previously described^15,16^. Cell suspensions (10^6^ cells/mL) were labeled with 2.5 μg/mL Hoechst 33,342 dye (Molecular Probes, Eugene, OR) in DMEM at 37 °C for 90 min with or without 50 μM verapamil (Sigma-Aldrich). To detect dead cells, we counterstained with 1.25 μg/mL propidium iodide. The cells were then analyzed with a FACS Vantage fluorescence-activated cell sorter (Becton Dickinson, Franklin Lakes, NJ) using dual wavelength analysis (blue, 424**–**444 nm; red, 675 nm long pass) after excitation with 350 nm UV light.

### Total RNA extraction

Total RNA was extracted from cells using the RNeasy Plus Mini Kit (Qiagen, Valencia, CA) according to the manufacturer’s instructions for QIAshredder (Qiagen). cDNA was generated by reverse transcription using ReverTra Ace α (Toyobo, Osaka, Japan).

### Real-time quantitative PCR analysis

Real-time PCR was carried out using a 7500 Real-Time PCR System with SDS RQ Study software ver. 2.0.6 (Applied Biosystems, Foster city, CA). cDNA templates were combined with SYBER Green premix with ROX (Takara, Shiga, Japan) to perform quantitative-PCR reactions. Each gene’s expression level was then compared relative to *GAPDH* or *HPRT* levels as internal standards. The primers are listed in Supplementary Table S8 online.

### Western blotting

Cellular proteins were extracted with lysis buffer (CelLytic M Cell Lysis Reagent; Sigma-Aldrich) with protease inhibitors (Protease Inhibitor Cocktail; Sigma-Aldrich). Equal amounts of proteins were loaded per lane, separated by SDS-PAGE and transferred to a nitrocellulose or polyvinylidene difluoride membrane. The membranes were incubated with diluted primary antibodies overnight at 4 °C, and then were incubated with horseradish peroxidase-linked secondary antibody. The blots were visualized by ECL using ChemiDoc XRS + System (Bio-Rad, Hercules, CA)^39^. The antibody dilutions and providers are listed in Supplementary Table S7 online.

### Sphere formation assay

Cells (1 × 10^3^) were plated in DMEM/F12 (Gibco, Waltham, MA) supplemented with B27 (Invitrogen), 20 ng/mL epidermal growth factor (EGF: Peprotech) and 20 ng/mL basic fibroblast growth factor (bFGF: Reprocell, Japan) on a low attachment 6-well plate (Corning, Kennebunk, ME). After 14 days, cells formed non-adherent spheres (P0). For serial passage, spheres were dissociated in Accutase (Innovative Cell Technologies Inc, San Diego, CA) and attained a single-cell suspension through a cell strainer with 40-μm nylon mesh. A single cell was plated in the above medium in each of 32 wells using 96-well, ultra-low attachment plates (Corning) and cultured for 2 weeks. Spheres were counted from the first through the fifth generation (P1-P5) under a microscope^40,41^. Spheres with diameters > 50 μm were counted.

### YBX2 siRNA experiment

*YBX2* siRNAs and negative control siRNA, which does not target any known cellular mRNA, were purchased from Invitrogen as follows: primer set *YBX2*, Human 2 Invitrogen, YBX2 stealth select RNAi 1 (YBX-HSS-181856), 2 (YBX-HSS-5192556), 3 (YBX-HSS-147268) and negative control (YBX-HSS-12935300). Transfection was performed with the Amaxa Cell Line Nucleofector Kit L (Lonza). After 24, 48 or 72 h, the cells were used for analyses.

### In vivo tumor formation assay

To initiate tumor formation, 1 × 10^6^ cells in 100 uL 1:1 DMEM and Matrigel (BD Matrigel Basement Membrane Matrix High Concentration; Becton Dickinson) solution were injected into the subcutaneous connective tissue of five-week-old nude mice (Balb/c nu/nu). The size of each tumor was measured every week. Tumor volume was calculated according to the following formula: tumor volume (mm^3^) = 0.5 × (major diameter) × (minor diameter)^2^. All mouse experiments were approved by the animal ethics committee of Kyusyu University (project number: A30-220) and complied with the guidelines of animal care of Kyusyu University. The study was carried out in compliance with the ARRIVE guidelines.

### Cell cycle analysis by flow cytometry

Cells were seeded into 6-well plates with 2 mL of culture medium. One day after seeding, the medium was replaced with 2 mL of serum-free culture medium. After 48 h, cells were refed with serum-containing culture medium. After 6, 12, 24, 36 and 48 h of culture, cells were collected and fixed in cold 70% ethanol. Then, cells were centrifuged (1500 × *g*, − 4 °C), washed with PBS and stained with propidium iodide solution (Sigma-Aldrich) containing RNase A (100 mg/mL, Qiagen). The cells were then used for flow cytometric analyses (BD FACSCalibur, Becton Dickinson) using ModFit LT analysis software ver. 3.3.11 (Verity Software House, Inc., Topsham, ME).

### Microarray

Microarray analysis was performed by CERI customer service (Saitama, Japan) in accordance with the instructions for One-Color Microarray-Based Gene Expression Analysis, ver 6.0. The integrity of the RNA was checked using an RNA 6000 Nano kit and 2100 Bioanalyzer (Agilent Technologies, Santa Clara, CA) as performed previously in our study^42^. Hybridization and washing were performed in accordance with the instructions of the manufacturer. Whole human Genome Array (4 × 44 K) ver. 2.0 (G4845A) (Agilent Technologies) was used to assess upregulated genes in a set of IK-YBX2 cells compared with that in mock cells. After washing, slides were scanned with an Agilent Microarray Scanner, and data were converted to gene expression data with Agilent Feature Extraction software ver. 10.7.1.1 Data were normalized by Agilent GeneSpring GX 11.5.1 software. Genes that were differentially expressed at least two-fold were considered significant.

The data associated with this paper is registered in GEO (study No GSE97511) at http://www.ncbi.nlm.nih.gov/geo/query/acc.cgi?acc=GSE97511.

Study No GSE97511.

### Immunocytochemistry

Cells (5 × 10^5^) were seeded in 8-well chamber slides. After 48 h, cells were fixed with 4% paraformaldehyde in PBS for 10 min. Cells were rinsed with PBS and incubated with PBS containing 0.25% Triton X-100 for 10 min to achieve permeabilization. Cells were incubated in Protein Block Serum Free (Dako) for 20 min. Cells were incubated with diluted primary antibodies for 90 min and then incubated with fluorescent-dye conjugated secondary antibodies for 30 min. Finally, cells were counterstained and mounted with ProLong Gold antifade reagent with DAPI (Invitrogen). All immunostaining steps were performed at room temperature. The antibodies used with dilution and provider are listed in Supplementary Table S7 online. Immunocytochemistry staining was detected under the BZ-X710 microscope (Keyence, Osaka, Japan), and images were merged using the Keyence BZ-X Analyzer software ver. 1.3.0.3 (Keyence).

### CT45 silencing by shRNA and CT45 rescuing

We established IK-YBX2 cells with the lentivirus system for stable knockdown of *CT45*. Small hairpin RNA (shRNA) targeted to *CT45* was delivered by transducing the cells with lentivirus carrying shRNA-encoding pLKO.1-puro vectors (Addgene, Cambridge, MA). We used puromycin (2 μg/mL) for selection of stable transduced cells. We constructed 5 kinds of shRNA for *CT45A5*. shCT45 sequences were designed using the 3′- untranslated region (*3-’UTR*)*.* We established *CT45* gene-*rescued* cells with the pLESIP sfi lentivector (EF1 promotor)-based production to explore gene functions. *CT45* cDNA was purchased from OriGene (Rockville, MD)*.* shRNA target sites and sequences are indicated in Supplementary Table S5 online.

### Chemosensitivity assay

Cells were seeded into 24-well plates at 2 × 10^4^ per well with 1 mL of culture medium. One day after seeding, the medium was replaced with 1 mL of medium. The following day, the medium was replaced with 1 mL of medium containing paclitaxel (Sigma-Aldrich), cisplatin (Bristol-Myers Squibb, New York, NY) or carboplatin (Wako, Osaka, Japan). Drugs were dissolved with dimethyl sulfoxide (Wako) before using. Cultured cells were counted 2, 4, 6 and 8 days after addition of drug. This assay was performed in triplicate.

### Statistical analysis

Data are represented as the means ± SD and were analyzed with Student’s *t*-test or with Fisher’s exact test. *P*-values for the one-sided test of less than 0.05 were considered statistically significant.

## Supplementary information

Supplementary information.

## Data Availability

All data generated or analyzed during this study are included in this article (and its supplementary information files).

## References

[1] Bray F (2018). Global cancer statistics 2018: GLOBOCAN estimates of incidence and mortality worldwide for 36 cancers in 185 countries. CA Cancer J. Clin..

[2] Yamagami W (2017). Clinical statistics of gynecologic cancers in Japan. J. Gynecol. Oncol..

[3] Morice P, Leary A, Creutzberg C, Abu-Rustum N, Darai E (2016). Endometrial cancer. The Lancet.

[4] Reya T, Morrison SJ, Clarke MF, Weissman IL (2001). Stem cells, cancer, and cancer stem cells. Nature.

[5] Visvader JE, Lindeman GJ (2008). Cancer stem cells in solid tumours: accumulating evidence and unresolved questions. Nat. Rev. Cancer.

[6] Bonnet D, Dick JE (1997). Human acute myeloid leukemia is organized as a hierarchy that originates from a primitive hematopoietic cell. Nat. Med..

[7] Lapidot T (1994). A cell initiating human acute myeloid leukaemia after transplantation into SCID mice. Nature.

[8] Al-Hajj M, Wicha MS, Benito-Hernandez A, Morrison SJ, Clarke MF (2003). Prospective identification of tumorigenic breast cancer cells. Proc. Natl. Acad. Sci..

[9] Singh SK (2003). Identification of a cancer stem cell in human brain tumors. Cancer Res..

[10] Dean M, Fojo T, Bates S (2005). Tumour stem cells and drug resistance. Nat. Rev. Cancer.

[11] Sugihara E, Saya H (2013). Complexity of cancer stem cells. Int. J. Cancer.

[12] Goodell MA (1996). Isolation and functional properties of murine hematopoietic stem cells that are replicating in vivo. J. Exp. Med..

[13] Zhou S (2001). The ABC transporter Bcrp1/ABCG2 is expressed in a wide variety of stem cells and is a molecular determinant of the side-population phenotype. Nat. Med..

[14] Kondo T, Setoguchi T, Taga T (2004). Persistence of a small subpopulation of cancer stem-like cells in the C6 glioma cell line. Proc. Natl. Acad. Sci..

[15] Kato K (2010). Endometrial cancer side-population cells show prominent migration and have a potential to differentiate into the mesenchymal cell lineage. Am. J. Pathol..

[16] Kato K (2011). Sodium butyrate inhibits the self-renewal capacity of endometrial tumor side-population cells by inducing a DNA damage response. Mol. Cancer Ther..

[17] Kohno K, Izumi H, Uchiumi T, Ashizuka M, Kuwano M (2003). The pleiotropic functions of the Y-box-binding protein, YB-1. BioEssays.

[18] Kohno Y (2006). Expression of Y-box-binding protein dbpC/contrin, a potentially new cancer/testis antigen. Br. J. Cancer.

[19] Tekur S, Pawlak A, Guellaen G, Hecht NB (1999). Contrin, the human homologue of a germ-cell Y-box-binding protein: cloning, expression, and chromosomal localization. J. Androl..

[20] Costa FF, Le Blanc K, Brodin B (2007). Concise review: cancer/testis antigens, stem cells, and cancer. Stem Cells.

[21] Gordeeva O (2018). Cancer-testis antigens: Unique cancer stem cell biomarkers and targets for cancer therapy. Semin. Cancer Biol..

[22] Yin B (2014). MAGE-A3 is highly expressed in a cancer stem cell-like side population of bladder cancer cells. Int. J. Clin. Exp. Pathol..

[23] Takeda R (2017). Identification and functional analysis of variants of a cancer/testis antigen LEMD1 in colorectal cancer stem-like cells. Biochem. Biophys. Res. Commun..

[24] Ma I, Allan AL (2011). The role of human aldehyde dehydrogenase in normal and cancer stem cells. Stem Cell Rev..

[25] Moore N, Lyle S (2011). Quiescent, slow-cycling stem cell populations in cancer: a review of the evidence and discussion of significance. J. Oncol..

[26] Chen YT (2005). Identification of cancer/testis-antigen genes by massively parallel signature sequencing. Proc. Natl. Acad. Sci. U. S. A..

[27] Yu CC, Hu FW, Yu CH, Chou MY (2016). Targeting CD133 in the enhancement of chemosensitivity in oral squamous cell carcinoma-derived side population cancer stem cells. Head Neck.

[28] Rahadiani N (2011). Expression of aldehyde dehydrogenase 1 (ALDH1) in endometrioid adenocarcinoma and its clinical implications. Cancer Sci..

[29] Nomura H (2019). Effect of taxane plus platinum regimens vs doxorubicin plus cisplatin as adjuvant chemotherapy for endometrial cancer at a high risk of progression: a randomized clinical trial. JAMA Oncol..

[30] Chen Y-T (2009). Cancer/testis antigen CT45: Analysis of mRNA and protein expression in human cancer. Int. J. Cancer.

[31] Chen YT (2011). Multiple cancer/testis antigens are preferentially expressed in hormone-receptor negative and high-grade breast cancers. PLoS ONE.

[32] Shang B (2014). CT45A1 acts as a new proto-oncogene to trigger tumorigenesis and cancer metastasis. Cell Death Dis..

[33] Zhou X (2013). Heterogeneous expression of CT10, CT45 and GAGE7 antigens and their prognostic significance in human breast carcinoma. Jpn. J. Clin. Oncol..

[34] Andrade VCC (2009). Frequency and prognostic relevance of cancer testis antigen 45 expression in multiple myeloma. Exp. Hematol..

[35] Wei X (2019). Cancer-testis antigen peptide vaccine for cancer immunotherapy: progress and prospects. Transl. Oncol..

[36] Allred DC, Bustamante MA, Daniel CO, Gaskill HV, Cruz AB (1990). Immunocytochemical analysis of estrogen receptors in human breast carcinomas. Evaluation of 130 cases and review of the literature regarding concordance with biochemical assay and clinical relevance. Arch. Surg..

[37] Kuramoto H, Tamura S, Notake Y (1972). Establishment of a cell line of human endometrial adenocarcinoma in-vitro. Am. J. Obstet. Gynecol..

[38] Yusuf N (2014). SPARC was overexpressed in human endometrial cancer stem-like cells and promoted migration activity. Gynecol. Oncol..

[39] Ohmaru-Nakanishi T (2018). Fibrosis in preeclamptic placentas is associated with stromal fibroblasts activated by the transforming growth factor-beta1 signaling pathway. Am. J. Pathol..

[40] Bisson I, Prowse DM (2009). WNT signaling regulates self-renewal and differentiation of prostate cancer cells with stem cell characteristics. Cell Res..

[41] Li SW (2015). The differential expression of OCT4 isoforms in cervical carcinoma. PLoS ONE.

[42] Inagaki T (2016). Up-regulation of lymphocyte antigen 6 complex expression in side-population cells derived from a human trophoblast cell line HTR-8/SVneo. Hum. Cell.

