# Supplementary material for: YBX2 and cancer testis antigen 45 contribute to stemness, chemoresistance and a high degree of malignancy in human endometrial cancer
Source: Sci Rep. 2021 Feb 18;11:4220. doi: 10.1038/s41598-021-83200-5 (PMC7893073; doi:10.1038/s41598-021-83200-5)
Supplement: Supplementary file 1 — Supplementary information. [file 41598_2021_83200_MOESM1_ESM.pdf]

Supplementary Information

YBX2 and cancer testis antigen 45 contribute to stemness, chemoresistance and a high degree of malignancy in human endometrial cancer

Izumi Suzuki[1,2*], Sachiko Yoshida[2*], Kouichi Tabu[3], Soshi Kusunoki[1], Yumiko Matsumura[2], Hiroto Izumi[4], Kazuo Asanoma[2], Hiroshi Yagi[2], Ichiro Onoyama[2], Kenzo Sonoda[5], Kimitoshi Kohno[6], Tetsuya Taga[3], Atsuo Itakura[1], Satoru Takeda[1], Kiyoko Kato[2*]

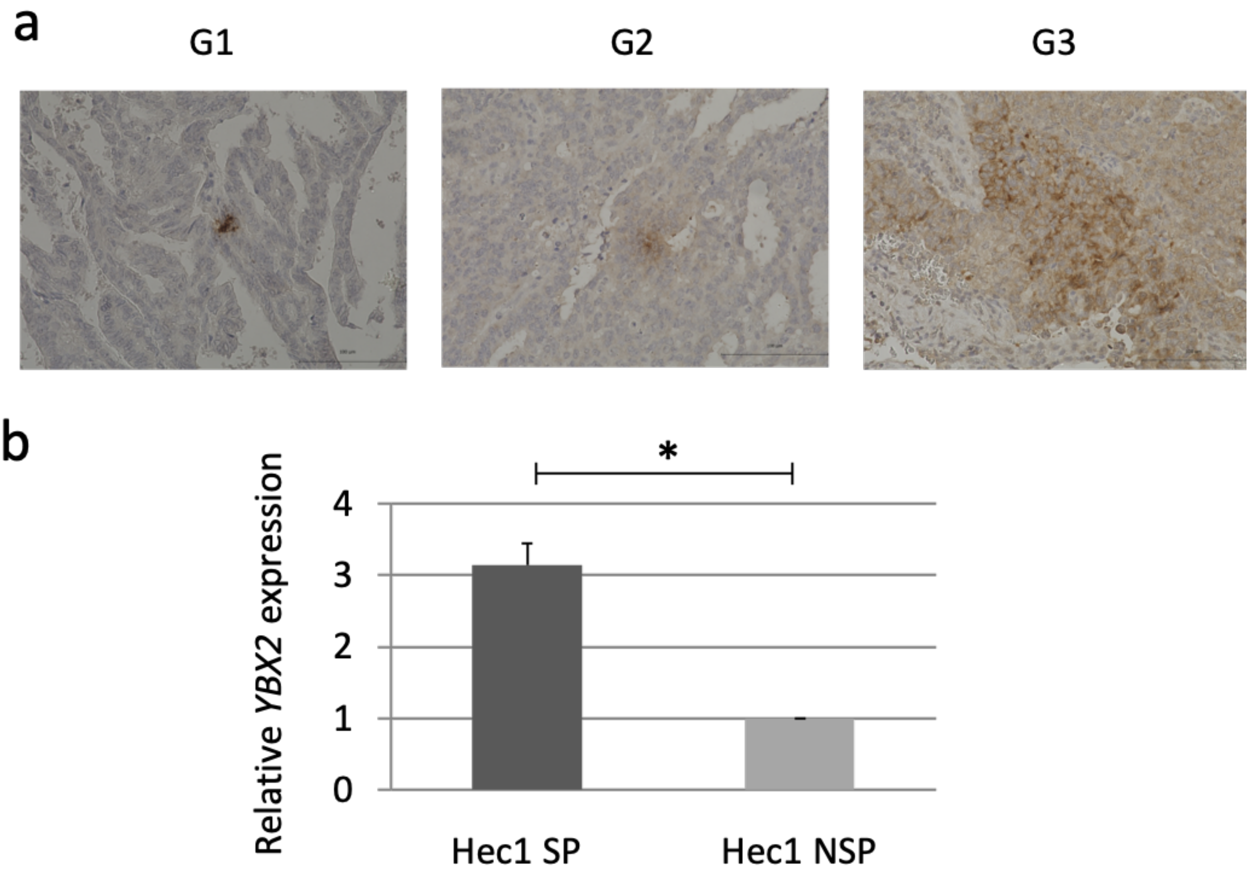

Supplementary Fig. S1: YBX2 expression in endometrial cancer tissue.

a) YBX2 expression in human endometrial cancer tissues (endometrioid carcinomas: **G1**, well-differentiated grade 1; **G2**, moderate-differentiated grade 2; **G3**, poorly differentiated grade 3) was investigated by immunohistochemistry. The entire slide was evaluated with the Allred scoring system in two categories (stain intensity and stain pattern) described in Supplementary Table S2. Representative results in each grade are shown.

b) The level of *YBX2* expression in Hec1-SP cells and Hec1-NSP cell was investigated by real-time PCR. *HPRT* levels were used as internal standards. Relative ratios are represented as the means ± SD from 3 independent experiments (*, $p < 0.05$).

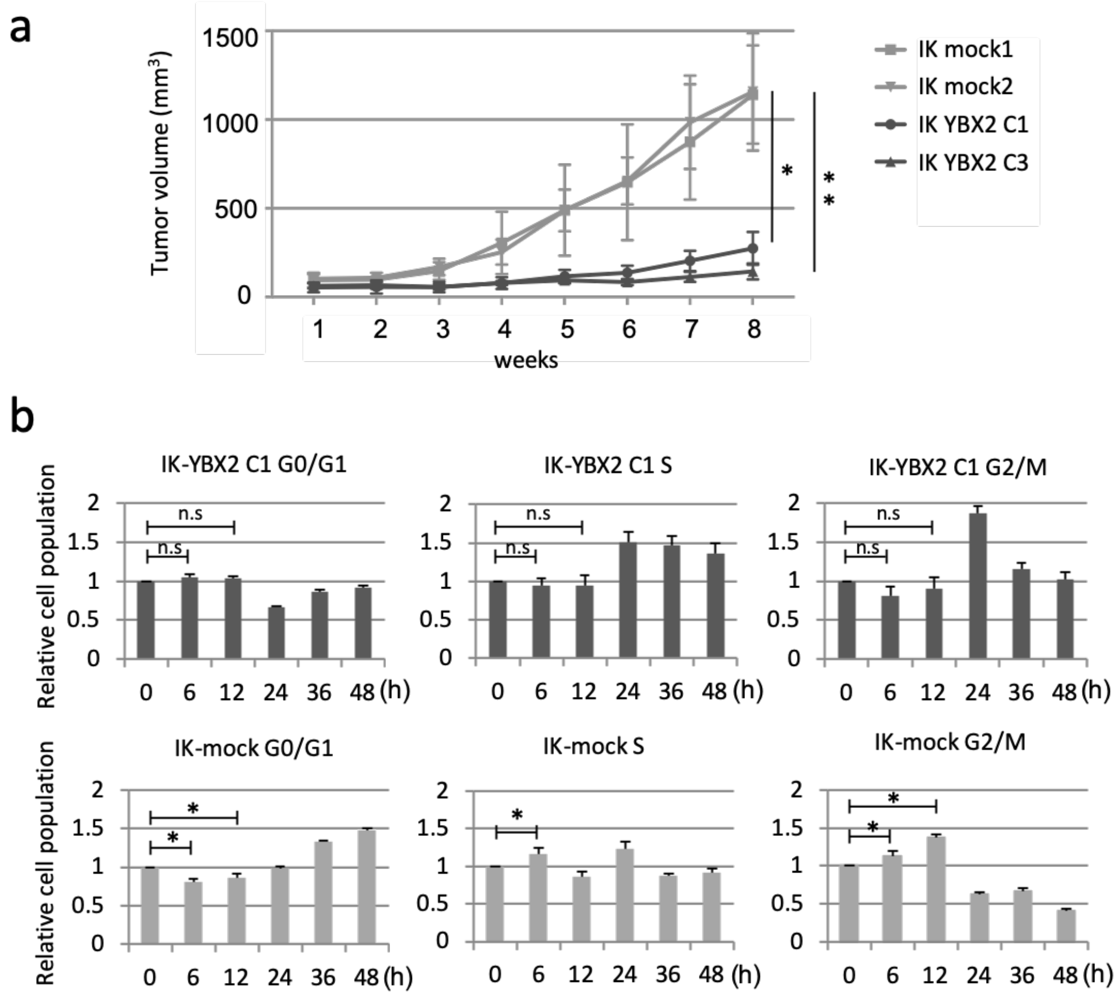

Supplementary Fig. S2: YBX2 suppressed cell growth and arrested the cell cycle by inhibition of the G1 to S phase transition.

a) IK-YBX2 C1, C3 cells or mock cells (1×10^6) were subcutaneously injected into the dorsal hypodermis of nude mice. Growth of the tumors from IK-YBX2 cells tended to be less than that of mock cells. Data represent the means ± SD, n = 10. (*, $p < 0.05$; **, $p < 0.01$).

b) YBX2 lengthened the cell cycle. The proportion of the IK-YBX2 C1 cell population in S phase and G2/M phase was not changed until 12 h of culture. In contrast, in the IK mock cell culture, the proportion of the population in S phase significantly increased at 6 h culture. Furthermore, that in G2/M phase was also significantly increased at 6 and 12 h (*, $p < 0.05$). Cells were stained with propidium iodide at the indicated time after 48 h serum-free culture. Sizes of cell populations averaged from 3 independent experiments with ± SD.

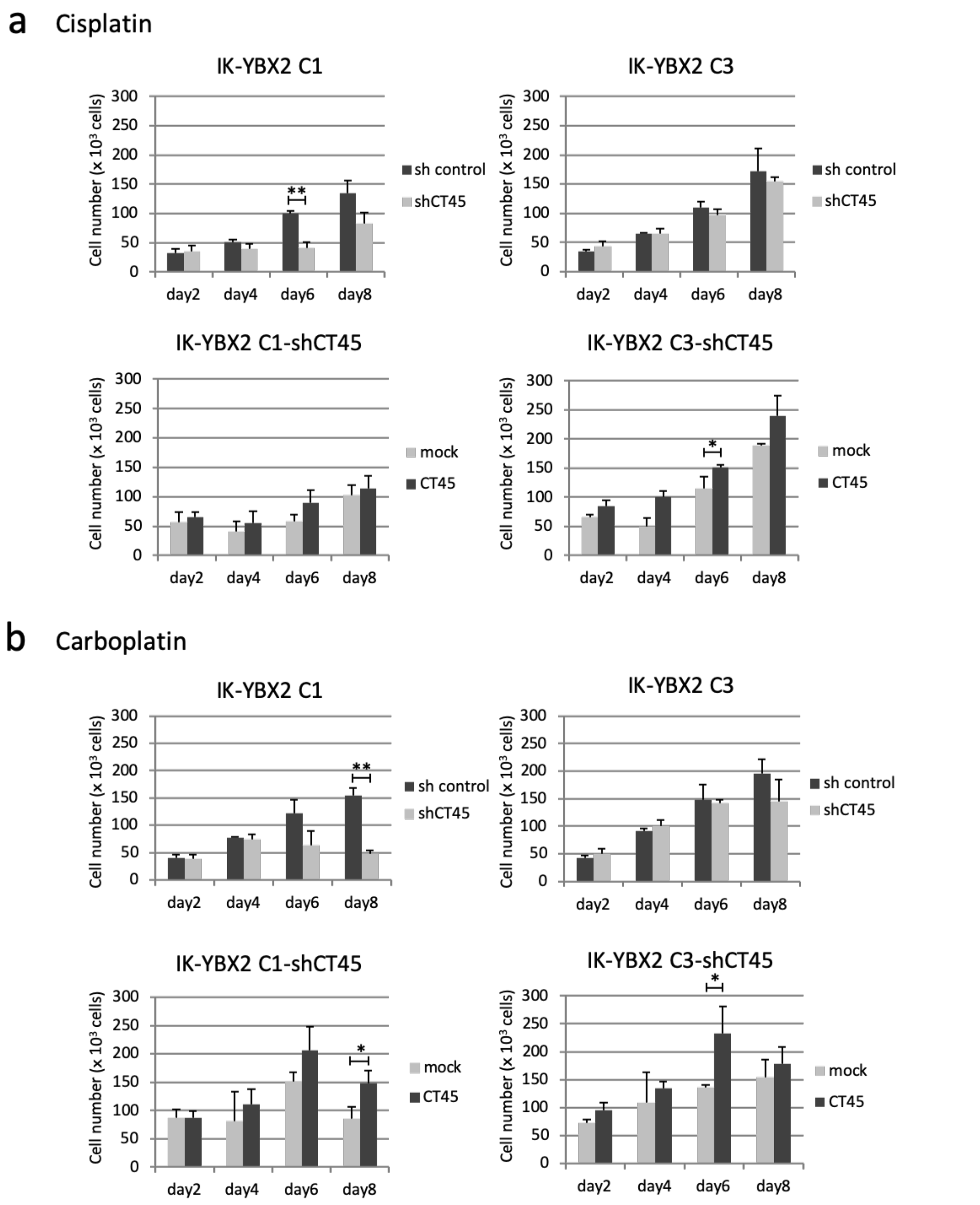

Supplementary Fig. S3: Chemosensitivity assays of IK-YBX2 C1 and C3-shCT45 cells vs. -sh control cells, and IK-YBX2-shCT45-CT45 rescued cells vs. mock cells.

a) The expression YBX2 and CT45 did not contribute to resistance to cisplatin.

b) The expression YBX2 and CT45 did not contribute to resistance to carboplatin.

(*, $p < 0.05$; **m $p < 0.01$)

Data represent the means ± SD from 3 independent experiments.

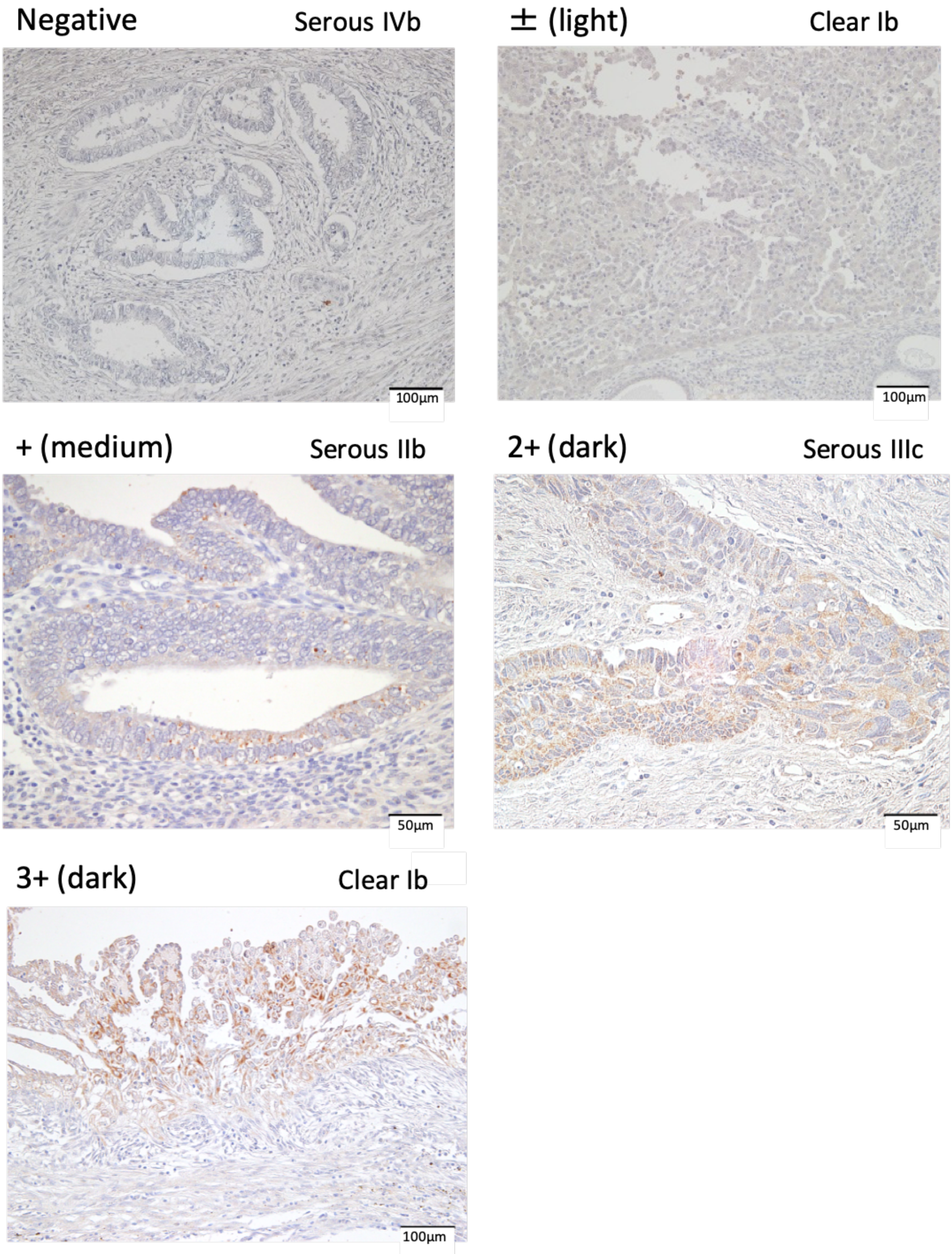

Supplementary Fig. S4: Expression pattern of CT45A5 in endometrial cancer tissues.

Expression of CT45A5 in endometrial cancer tissues was investigated by immunohistochemistry. The entire slide was evaluated with the Allred scoring system in which 2 categories (stain intensity and stain pattern) were evaluated as described in Supplementary Table S2.

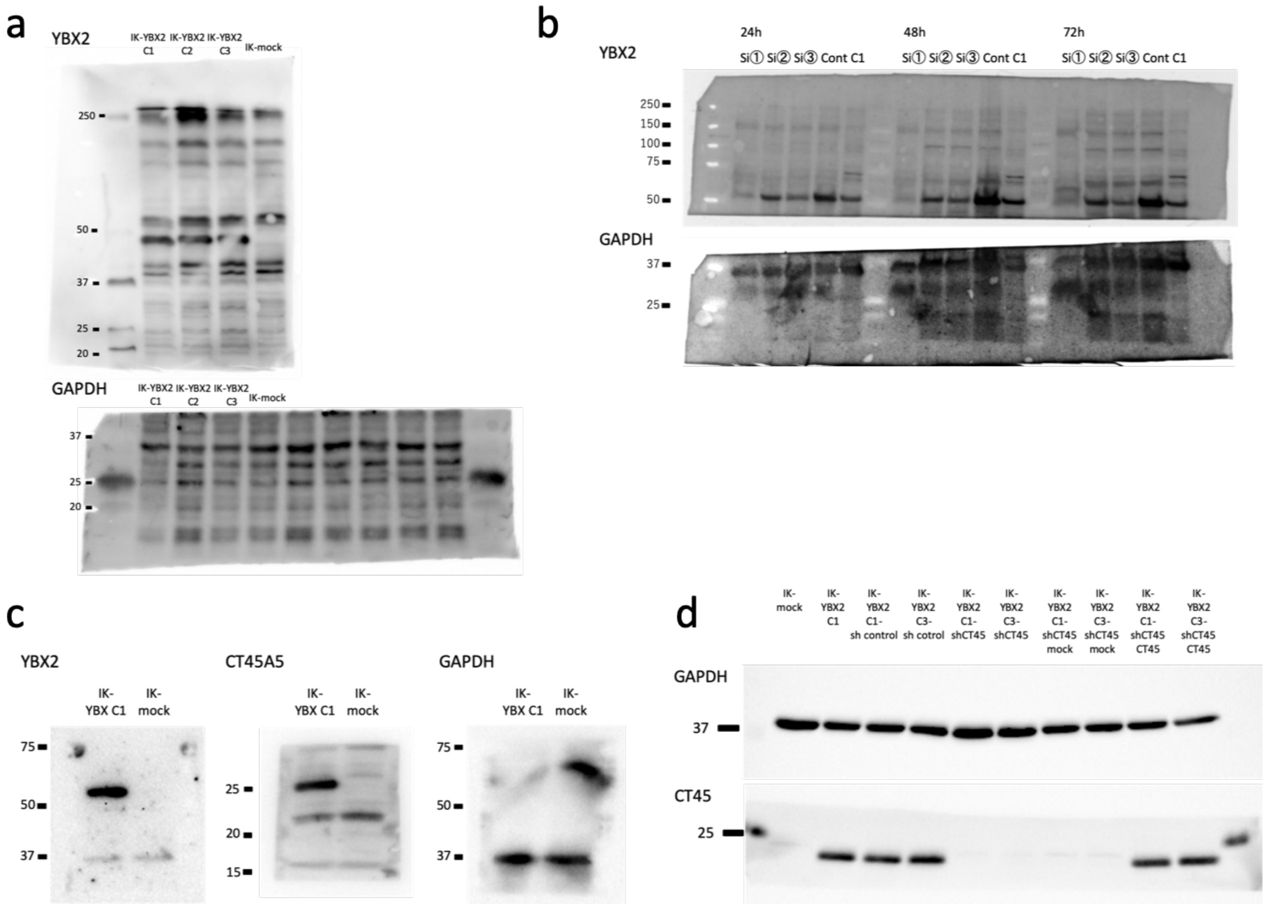

Supplementary Fig. S5: Full length blots of Western blotting.

Full length blots of the Western blots shown in a) Fig. 1a, b) Fig. 2a, c) Fig. 3c, d) Fig. 4a.

Supplementary Table S1. Patient characteristics (YBX2)

	Endometrioid		
	G1	G2	G3
stage			
I	5	3	5
II	3	2	1
III	1	3	3
IV	1	2	1
No. of patients	10	10	10

Supplementary Table S2. Allred scoring system

Allred scoring system: two categories (stain intensity and stain pattern)
[intensity score (IS)]: 0, 1, 2, and 3
(for none, light, medium, or dark staining)
[proportional score (PS)]: no stain = 0;
≤1/100 cells stained = 1;
≤1/10 cells stained = 2;
≤1/3 cells stained = 3;
≤2/3 cells stained = 4;
all cells stained = 5.
Any score above 2 was considered positive.

Supplementary Table S3. Expression pattern of YBX2 in endometrial cancer tissue

grading	positive	negative	positive/total(%)	
G1	1	9	10	
G2	3	7	30	n.s *
G3	6	4	60	

Expression of YBX2 in endometrial cancer tissues was investigated by immunohistochemistry. Patient characteristics are shown in Supplementary Table S1. The entire slide was evaluated by the Allred scoring system in which 2 categories (stain intensity and stain pattern) were evaluated as described in Supplementary Table S2.

There was no significant difference between G1 and G2. The positive level of YBX2 in G3 was significantly higher than that in the G1 group. (*: $p < 0.05$).

Supplementary Table S4. The top ten up-regulated genes in IK-YBX2 C1 cells compared with in mock cells

Gene ID	Symbol	Log_2 ratio	Fold-Change
Hs.535081	***CT45A5***	9.571	760.566
Hs.643864	***SPON1***	7.142	141.279
Hs.459642	***CACNA1H***	6.955	124.058
Hs.654428	***HLA-DMB***	6.122	69.643
Hs.301350	***FXYD3***	5.192	36.558
Hs.446962	***C1orf194***	5.052	33.170
Hs.59761	***DAPL1***	4.708	26.130
Hs.177841	***BHLHE41***	4.564	23.281
Hs.183109	***MAOA***	4.541	23.281
Hs.655915	***C7orf29***	4.498	22.595

Supplementary Table S5. List of the shRNA target sites and sequences

**shRNA name**	**Target**	**Target site**	**shRNA sequence**<br>**(cloned into Age Ⅰ and EcoR Ⅰ)**
**Control shRNA**	None	CCTAAGGTTAA<br>GTCGCCCTCG	5'-<br>accggtCCTAAGGTTAAGTCGCCCTCGC<br>TCGAGCGAGGGCGACTTAACCTTAGG<br>TTTTTTgaattc-3'
**shCT45**<br>**(*3’UTR*)**	CT45<br>A1, A3,<br>A5, A6,<br>A7, A10	GCATAATCTCG<br>TTAATGATTG	5’-<br>accggtGCATAATCTCGTTAATGATTGCT<br>CGAGCAATCATTAACGAGATTATGCTT<br>TTTTGGAAgaattc-3'

Supplementary Table S6. Patient characteristics (CT45A5)

C	Endometrioid			Serous	Clear cell
	**G1**	**G2**	**G3**		
**stage**					
**I**	**54**	**15**	**14**	**6**	**8**
**II**	**3**	**3**	**2**	**-**	**-**
**III**	**4**	**4**	**6**	**4**	**4**
**IV**	**1**	**-**	**-**	**2**	**-**
**No. of patients**	**62**	**22**	**22**	**12**	**12**

Supplementary Table S7. List of the antibodies used in the study

Antibody	Species	Dilution	Source
YBX2 (IHC/WB)	rabbit polyclonal	1:100 / 1:1000 (IHC/WB)	Gift from Dr. Kohno[18]
YBX2 (ICC)	mouse polyclonal	1:50	Novus Biologicals (Littleton, CO)
CT45A5 (WB)	rabbit polyclonal	1:1000	MBL (Nagano, Japan)
CT45(whole) (IHC/WB/ICC)	goat polyclonal	1:100 /1:1000 /1:50 (IHC/WB/ICC)	Santa Cruz Biotechnology (Santa Cruz, CA)
GAPDH	rabbit polyclonal	1:1000	Santa Cruz Biotechnology
ALDH1	mouse monoclonal	1:500	Becton Dickinson (Franklin Lakes, NJ)/ Santa Cruz Biotechnology
Secondary antibody (WB)	anti-rabbit IgG	1:4000	Sigma-Aldrich (St. Louis, MO)
Secondary antibody (WB)	anti-mouse IgG	1:3000	Sigma-Aldrich
Secondary antibody (ICC)	Alexa Fluor 488 goat anti-mouse IgG	1:500	Invitrogen (Carlsbad, CA)
Secondary antibody (ICC)	NorthernLights anti-goat IgG-NL557	1:200	R&D systems (Minneapolis, MN)

IHC: Immunohistochemistry, WB: Western Blotting, ICC: Immunocytochemistry

Supplementary Table S8. List of the primers used in the study

primer		primer sequence
GAPDH	forward	5’-GAAGGTGAAGGTCGGAGTC-3’
	reverse	5’-GAAGATGGTGATGGGATTTC-3’
HPRT	forward	5’-GTCAAGAGCATATCCTACAACAAAC -3’
	reverse	5’-GGCAGTATAATCCAAAGATGGTCAA -3’
YBX2	forward	5'-AGTTTCTGCGCAGCGTTGG-3'
	reverse	5'-GGATGAATCGGCGGGACTTAC-3'
ALDH1	forward	5'-TGTTAGCTGATGCCGACTTG -3'
	reverse	5'-TTCTTAGCCCGCTCAACACT -3'
CT45A5	forward	5'-AAGGTGGCTGTAGATCCTGAAACTG-3'
	reverse	5'-ATCCAATTGGCTGGGTGGAA-3'
